# An Organic Solvent-Assisted Intercalation and Collection (OAIC) for Ti_3_C_2_T_x_ MXene with Controllable Sizes and Improved Yield

**DOI:** 10.1007/s40820-021-00705-4

**Published:** 2021-09-05

**Authors:** Danyao Qu, Yingying Jian, Lihao Guo, Chen Su, Ning Tang, Xingmao Zhang, Wenwen Hu, Zheng Wang, Zhenhuan Zhao, Peng Zhong, Peipei Li, Tao Du, Hossam Haick, Weiwei Wu

**Affiliations:** 1 School of Advanced Materials and Nanotechnology, Xidian University, Xi’an, Shaanxi 710126 People’s Republic of China; 2 School of Aerospace Science and Technology, Xidian University, Xi’an, Shaanxi 710126 People’s Republic of China; 3 Department of Chemical Engineering and Russell Berrie Nanotechnology Institute, Technion-Israel Institute of Technology, 3200003 Haifa, Israel

**Keywords:** Two-dimensional materials, MXenes, Controllable sizes, High yield

## Abstract

A good method of synthesizing Ti_3_C_2_T_x_ (MXene) is critical for ensuring its success in practical applications, e.g., electromagnetic interference shielding, electrochemical energy storage, catalysis, sensors, and biomedicine. The main concerns focus on the moderation of the approach, yield, and product quality. Herein, a modified approach, organic solvent-assisted intercalation and collection, was developed to prepare Ti_3_C_2_T_x_ flakes. The new approach simultaneously solves all the concerns, featuring a low requirement for facility (centrifugation speed < 4000 rpm in whole process), gram-level preparation with remarkable yield (46.3%), a good electrical conductivity (8672 S cm^−1^), an outstanding capacitive performance (352 F g^−1^), and easy control over the dimension of Ti_3_C_2_T_x_ flakes (0.47–4.60 μm^2^). This approach not only gives a superb example for the synthesis of other MXene materials in laboratory, but sheds new light for the future mass production of Ti_3_C_2_T_x_ MXene.

**Supplementary Information:**

The online version contains supplementary material available at 10.1007/s40820-021-00705-4.

## Highlights

- An organic solvent-assisted intercalation and collection (OAIC) approach for preparing Ti_3_C_2_T_x_ (MXene) was developed.
- The features of OAIC approach are the low requirement for facility, gram-level preparation with remarkable yield and easy control over the dimension of Ti_3_C_2_T_x_ flakes.
- The Ti_3_C_2_T_x_ flakes prepared by OAIC approach showed the outstanding capacitive and rate performance.

**Supplementary Information:**

The online version contains supplementary material available at 10.1007/s40820-021-00705-4.

## Graphical abstract

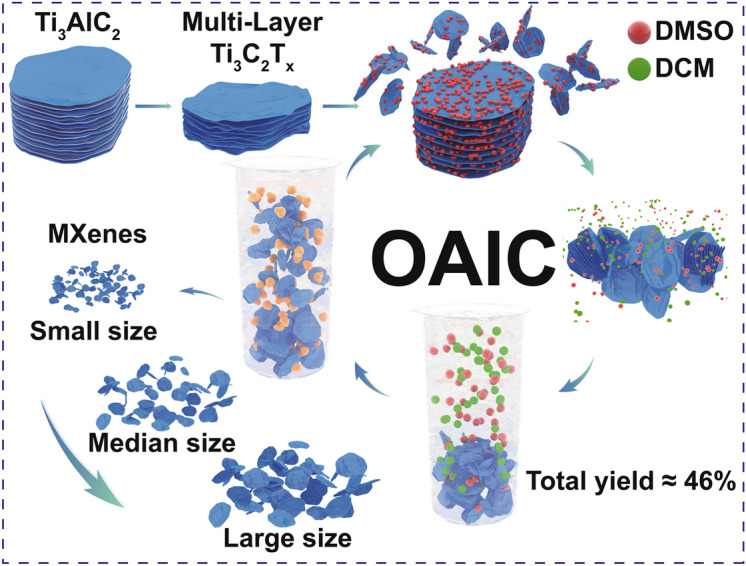

## Introduction

Ti_3_C_2_T_x_ (MXene), a family of cutting-edge two-dimensional (2D) nanomaterials [1, 2], has a wide range of potential applications, including electromagnetic interference shielding [3], electrochemical energy storage [4, 5], catalysis [6], sensors [7], biomedicine [8], etc. As the building block of these important applications, more attention has currently been put on synthetic methods that can deliver the large-scale demand of materials for real industrial applications. A good synthetic method of nanomaterials requires good controllability, high yield, low cost, green processes, and safety, which are the main factors in deciding that a synthetic method can leave laboratory demonstrations and go to mass production.

**Fig. 1:**
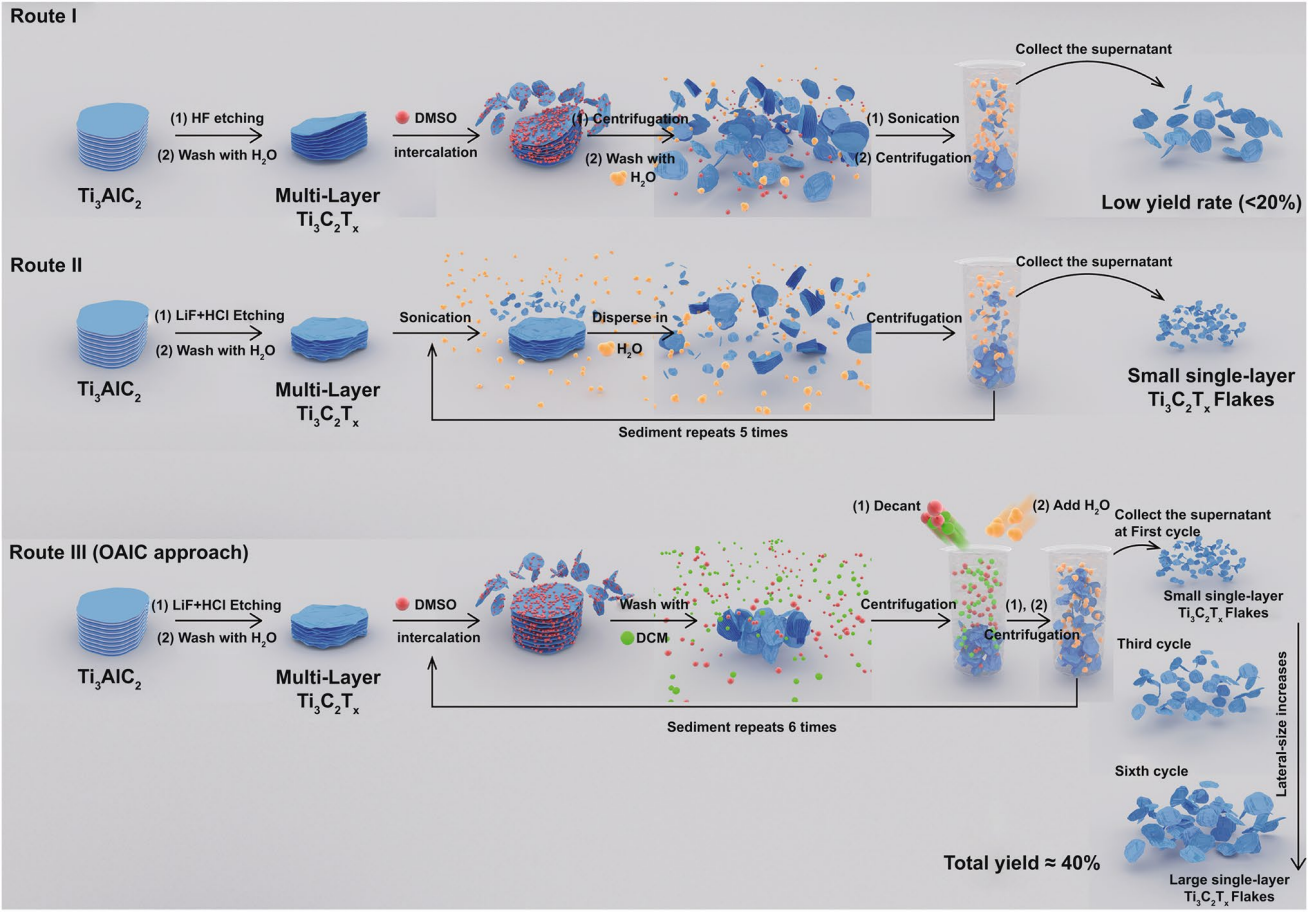
Schematic flow process for preparing Ti_3_C_2_T_x_ flakes by Route I (HF etching followed by DMSO intercalation), Route II (LiF/HCl etching, followed by sonication) and Route III (organic solvent-assisted intercalation and collection approach)

Similarly, the continuous development of novel synthetic methods is present throughout the whole road-map of MXene materials [9]. Initially, Ti_3_C_2_T_x_ flakes were prepared by two steps including etching the Ti_3_AlC_2_ (MAX phase) by concentrated hydrofluoric acid (HF) and then intercalating multilayered sheets with organic molecules, e.g., hydrazine, urea, and dimethyl sulfoxide (DMSO) **(**Route I in Fig. 1) [10]. However, the utilization of hazardous and toxic HF not only makes the operations risky, but the low yield (˂20%) [11] further suggests it could not possibly use in mass production. Later, a more moderate etching system was reported using the fluoride salt, LiF, mixed with HCl. In this system, HF is formed in situ with lithium ions intercalated during the etching process, resulting in Ti_3_C_2_T_x_ “clay.” After sonication in water, single Ti_3_C_2_T_x_ flakes with submicron lateral-size could be isolated (Route II in Fig. 1) [12]. The pros and cons are the avoidance of having to use HF, and the small size of Ti_3_C_2_T_x_ flakes with low yield, respectively. Recently, some further modifications have been developed to improve the yield and/or increase the size of Ti_3_C_2_T_x_ flakes [13–17]. However, a method with a perfect trade-off that meets all the aforementioned requirements, *inc.*, good controllability, high yield, low cost, green process, and safety, has to been achieved and remains challenging.

In this work, a modified approach, organic solvent-assisted intercalation and collection (OAIC), is reported to prepare Ti_3_C_2_T_x_ flakes in gram-level with enhanced yield (46.3%) and improved quality (area reaches *ca.* 4.60 μm^2^) through a facile approach featuring sonication-free and high-speed centrifugation-free (< 4000 rpm) methods. More importantly, the Ti_3_C_2_T_x_ flakes of different sizes can be obtained in different production cycles instead of gradient centrifugation. The Ti_3_C_2_T_x_ flakes with median size show the outstanding capacitive and rate performance. To our best knowledge, the OAIC approach that simultaneously meets aforementioned features of a good synthetic method is a novel procedure, which could shed new light on the mass production of other MXene materials in the future.

## Experimental Section

### Materials

LiF (Sinopharm Chemical Reagent Co., Ltd.), HCl (Sinopharm Chemical Reagent Co., Ltd.), Ti_3_AlC_2_ (Forsman Scientific, Beijing, Co., Ltd.), DMSO (Aladdin Bio-ChemTechnology Co., Ltd.), dichloromethane (DCM, Sinopharm Chemical Reagent Co., Ltd.), and H_2_SO_4_ (Sinopharm Chemical Reagent Co., Ltd.) are used as purchased without further purification.

### Characterizations

The structure and microtopography of Ti_3_C_2_T_x_ were taken with field-emission scanning electron microscopy (FESEM, FEI Apreo HiVac, 5 kV) and transmission electron microscopy (TEM, JEOL JEM-2100F, 200 kV). The crystallographic information was examined by an X-ray diffraction (XRD, Bruker D8 Advance X-ray diffractometer) under filtered Cu-Kα radiation (40 kV and 40 mA, *λ* = 0.15418 nm), with a step scan of 0.01°, a 2*θ* range of 5–65°, and a step time of 2 s. The element compositions of Ti_3_C_2_T_x_ were analyzed by the X-ray photoelectron spectroscopy (XPS, Kratos-AXIS Supra) and energy-dispersive spectroscopy (EDS, X-Max N 80 T). The atomic force microscope (AFM) image was obtained by Asylum Research AFM. Raman spectroscopy was obtained by a spectrometer model of Renishaw-inVia. The ultraviolet–visible diffuse reflectance spectra (UV–vis DRS) test is obtained by a Hitachi UH4150 spectrophotometer.

### Synthesis of Ti_3_C_2_T_x_

The Ti_3_C_2_T_x_ flakes synthesized via Route II (the products are denoted as **S-Ti**_**3**_**C**_**2**_**T**_**x**_.), which by selective etching of Al atoms from Ti_3_AlC_2_ using HCl/LiF etchant followed as previous report [12]. For OAIC approach, all synthetic procedure of gram-level preparation of Ti_3_C_2_T_x_ was recorded in Movie S1 (Supporting Information). The difference between routine preparation (the products are denoted as **O-Ti**_**3**_**C**_**2**_**T**_**x**_) described here and gram-level preparation is nothing, but the amount of raw Ti_3_AlC_2_ and all chemical reagents is enlarged ten-fold. Specifically, for routine preparation, concentrated HCl (35% solution) was added to DI water to prepare the 9 M HCl solution (20 mL total) in a Teflon reactor (Step 1 in Movie S1). 1.6 g LiF was slowly added to this solution and stirred for 10 min with a magnetic Teflon stir bar to dissolve the salt (Step 2). Then, 1 g of Ti_3_AlC_2_ powders was slowly added into prepared HCl/LiF solution over the course of 10 min to avoid initial overheating of the solution. The reaction mixture was then held at 35 ℃ for 24 h (Step 3). Next, the mixture was washed through at least 4 times of DI water addition, centrifugation (4000 rpm), and decanting, until the supernatant reached to neutral (pH≈7) (Step 4). The critical step of OAIC approach is to add multi-layer Ti_3_C_2_T_x_ to DMSO at a ratio of 1 g to 20 mL, and stirring at room temperature for 3 h (Step 5), and then, DCM (V_DCM_:V_DMSO_ = 1:1) was added to the mixture for completely removing DMSO. The O-Ti_3_C_2_T_x_ products were obtained after the centrifugation at speed of 2,000 rpm for at least three times (Step 6). As shown in Figure S1a, the supernatant was nearly clear, indicating little products loss in this step. The clay-like products were obtained by vacuum drying at room temperature for 2 h to remove the residual DCM (Step 7). Then, the DI water was added, vortex shock dispersed, and centrifugated at 3,500 rpm for 30 min to separate the supernatant and sediment (Step 8). The synthesized O-Ti_3_C_2_T_x_ flakes dispersed in water homogeneously, which could be stored for one week without obvious precipitation (Fig. S1b), indicating good dispersion stability. The Tyndall scattering effect cloud be observed depending on green laser passing through the colloidal solution (Fig. S1b), also indicating the dispersion stability. Taking the supernatant for freeze drying to obtain O-Ti_3_C_2_T_x_ products, which named as “O-Ti_3_C_2_T_x_-1” (Step 9). The sediments in step 8 were repeated by step 5 to step 9 for other five cycles to extract the products, which named as “O-Ti_3_C_2_T_x_-2” to “O-Ti_3_C_2_T_x_-6.” The O-Ti_3_C_2_T_x_ dispersions of each cycle were shown in step 8 in Movie S1. The final O-Ti_3_C_2_T_x_ products were weighted for the calculation of the yield. In the gram-level preparation, the volumes of dispersion in step 8 in each cycle were accurately measured which denoted as *V*_dis_. Then, the dispersion of each cycle was extracted for five parts with 5 mL of each part. After freeze drying, the O-Ti_3_C_2_T_x_ products of each part were weighted and denoted as *m*_*1*_ to *m*_*5*_. The mass concentration of the dispersion can be calculated by Eq. 1:

1 $$c = \frac{{\overline{m}}}{V}$$

where $\overline{m}$ is the average mass of five parts, *V* is 5 mL. So far, the mass of O-Ti_3_C_2_T_x_ products in dispersion of each cycle (denote as *m*_dis_) can be calculated by Eq. 2:

2 $$m_{dis} = cV_{dis}$$

which are summarized in Table S2. The yield of gram-level preparation was calculated by total mass of O-Ti_3_C_2_T_x_ products in the dispersion dividing by the mass of Ti_3_AlC_2_ powders (10 g). Therefore, the O-Ti_3_C_2_T_x_ products with controlled sizes can be finally obtained in the form of both solid and aqueous dispersion according to the requirements of sample preparation for different applications.

### Measurement of the Average Area and Lateral Size of Ti_3_C_2_T_x_ Flakes

TEM-derived area distributions were determined by measuring the area of 100 flakes using ImageJ analysis software. Histograms were created using 50 bins from 0 to 10 μm^2^, and log-normal distributions were fit. The average value of Ti_3_C_2_T_x_ flakes area and SD was extracted from normal distribution fitting. The representative TEM images and Ti_3_C_2_T_x_ flakes area log-normal distributions of products from different cycles are shown in Fig. S6a, b. The corresponding lateral sizes of Ti_3_C_2_T_x_ flakes were measured by a similar approach, which listed in Fig. S6.

### Preparation of Free-standing Ti_3_C_2_T_x_ Films

The free-standing Ti_3_C_2_T_x_ films were fabricated through vacuum filtration of the produced Ti_3_C_2_T_x_ dispersion from different cycles on a polyvinyl difluoride (PVDF) membrane (0.45 μm pore size). The Ti_3_C_2_T_x_ films were dried in a vacuum system and peeled off from the PVDF membrane. Digital photograph of free-standing O-Ti_3_C_2_T_x_ film is shown in Figure S1c. The thickness of the obtained Ti_3_C_2_T_x_ films was measured by micrometer caliper.

### Conductivity Measurements

The electrical conductivity of Ti_3_C_2_T_x_ films was tested by a four-point probe mapping system (280SI, Four Dimensions Inc., the USA). The Ti_3_C_2_T_x_ films were cut into circular shape with diameter of 15 mm. Electronic conductivity σ (S cm^−1^) was calculated by Eq. 3:

3 $$\sigma { = }\frac{1}{{R_{s} \times d}}$$

where *R*_*s*_ (Ω sq^−1^) is the average sheet resistance measured by four-point probe mapping system, and *d* is the thickness of Ti_3_C_2_T_x_ film.

### Electrochemical Measurements

All electrochemical measurements were performed using a CHI660E potentiostat (Chenhua Instruments, Shanghai, China). Three-electrode electrochemical tests were performed in a plastic Swagelok cell (Fig. S1d) where glassy carbon electrodes were used as current collectors for both the working and the counter electrodes [12, 18]. The prepared Ti_3_C_2_T_x_ films were punched to the desired size and directly used as the working electrode without addition of any binder. The free-standing overcapactive activated carbon electrode was used as the counter electrode, which was prepared by mixing 95 wt.% of the activated carbon (YP-50, Kuraray, Japan) with 5 wt% PTFE (60 wt% dispersion in H_2_O, Sigma-Aldrich) in ethanol [18]. The Ag/AgCl electrode in 1 M KCl was used as the reference electrode. Two pieces of Celgard paper (3501) were used as the separator between the working electrode and the counter electrode. The deaerated 3 M H_2_SO_4_ was used as the electrolyte (Fig. S1e).

The cyclic voltammetry (CV) was performed at different scan rates ranging from 2 mV s^−1^ to 10 V s^−1^ with a working potential window of 0.9 V (− 0.7 to 0.2 V vs. Ag/AgCl). Gravimetric capacitances were calculated by integration of the discharge curves in the CV plots using Eq. 4:

4 $$C = \frac{\int I dt}{{m\Delta V}}$$

where C is the gravimetric capacitances (F g^−1^), I is the discharging current, m is the mass of the Ti_3_C_2_T_x_ films working electrode, and ∆V is the voltage scan window. Galvanostatic charging and discharging (GCD) was performed at the potential window of 0.9 V (− 0.7 to 0.2 V vs. Ag/AgCl). The O-Ti_3_C_2_T_x_-3 film electrode was used to measure long-term cyclability at 10 A g^−1^. The GCD was cycled for 10,000 cycles with a 0.9 V potential window (− 0.7 to 0.2 V vs. Ag/AgCl), and gravimetric capacitance in stability test was calculated from the discharging curve. Electrochemical impedance spectroscopy (EIS) was performed at the open circuit potentials of the cells, with a 10 mV amplitude and frequencies ranging from 100 mHz to 100 kHz.

## Result and Discussion

### Features of the OAIC Approach

Among the various synthetic methods of Ti_3_C_2_T_x_ flakes, the traditional 2-step method involves etching the Al atomic layer selectively in the MAX phase by HF, and then obtaining a single layer of Ti_3_C_2_T_X_ intercalated by the organic reagent, DMSO, illustrated as Route I in Fig. 1. DMSO can be removed by DI water washing and high-speed centrifugation due to the miscibility of DMSO in water. However, due to the high affinity of DMSO, water and Ti_3_C_2_T_x_ flakes, even after centrifugation of 10,000 rpm, large amounts of Ti_3_C_2_T_x_ flakes remain dispersed in the supernatant, causing weight loss of the products. In addition, the synthetic method of Route I has high safety risks, and unfavorable hazardous chemicals, which are big hindrances to mass production. Route II is the currently most used method for preparing Ti_3_C_2_T_x_ flakes which avoids HF addition. Exfoliations are achieved under strong sonication to obtain the flakes. However, violent mechanical energy during sonication drastically reduces flakes size which is unsatisfied in applications requiring good conductivity and structural integrity [19]. Otherwise, the minimally intensive layer delamination (MILD) approach yields large-size Ti_3_C_2_T_x_ flakes, but the vital yield is still unmentioned [3, 20]. Therefore, lessons are learned from Route I and Route II, including several aspects, i.e., avoiding the use of HF, strong sonication, high-speed centrifugation, and weight loss in the decant after centrifugation. The OAIC approach (Route III) is a modified method which combines a part of Route I and Route II, of which features include—but not limited—in the following ways. First, the LiF/HCl etching process is the same as Route II, which meets the moderate etching process. Second, intercalation is reinforced by DMSO, which is similar as most intercalating chemicals in Route I. Third, the well-known “similia similibus solventur” principle is adopted to remove the residual DMSO by DCM because the magnitude of solvent–solvent interactions is much larger than solvent-nanoflakes interactions [21]. In addition, the low boiling point of DCM (39.75 °C at 760 mmHg) is what help DCM to volatilize by vacuum drying. Most significantly, inspired from the fabrication of graphene and Ti_3_C_2_T_x_ macro-fibers [22, 23], DCM washing efficiently restrain large weight loss in Route I which is caused by the step of water adding for removing DMSO because of the strong interactions between water and Ti_3_C_2_T_x_ flakes. After adding DI water, Ti_3_C_2_T_x_ flakes can be brought down from the supernatant by centrifugation (3500 rpm). The sediment can have DMSO added to further intercalate the residues, in which more Ti_3_C_2_T_x_ flakes can be extracted after DCM washing and centrifugation in water to enhance the yield. This step can be cycled at least 6 times; each cycle accumulates more Ti_3_C_2_T_x_ flakes with increased size in gradients, which is the fourth feature of OAIC approach and the first reported feature among all other approaches. All details of OAIC approach were described and discussed in the experimental section, Movie S1 and supporting information.

### Morphology of Ti_3_C_2_T_x_ Flakes Prepared by OAIC

**Fig. 2:**
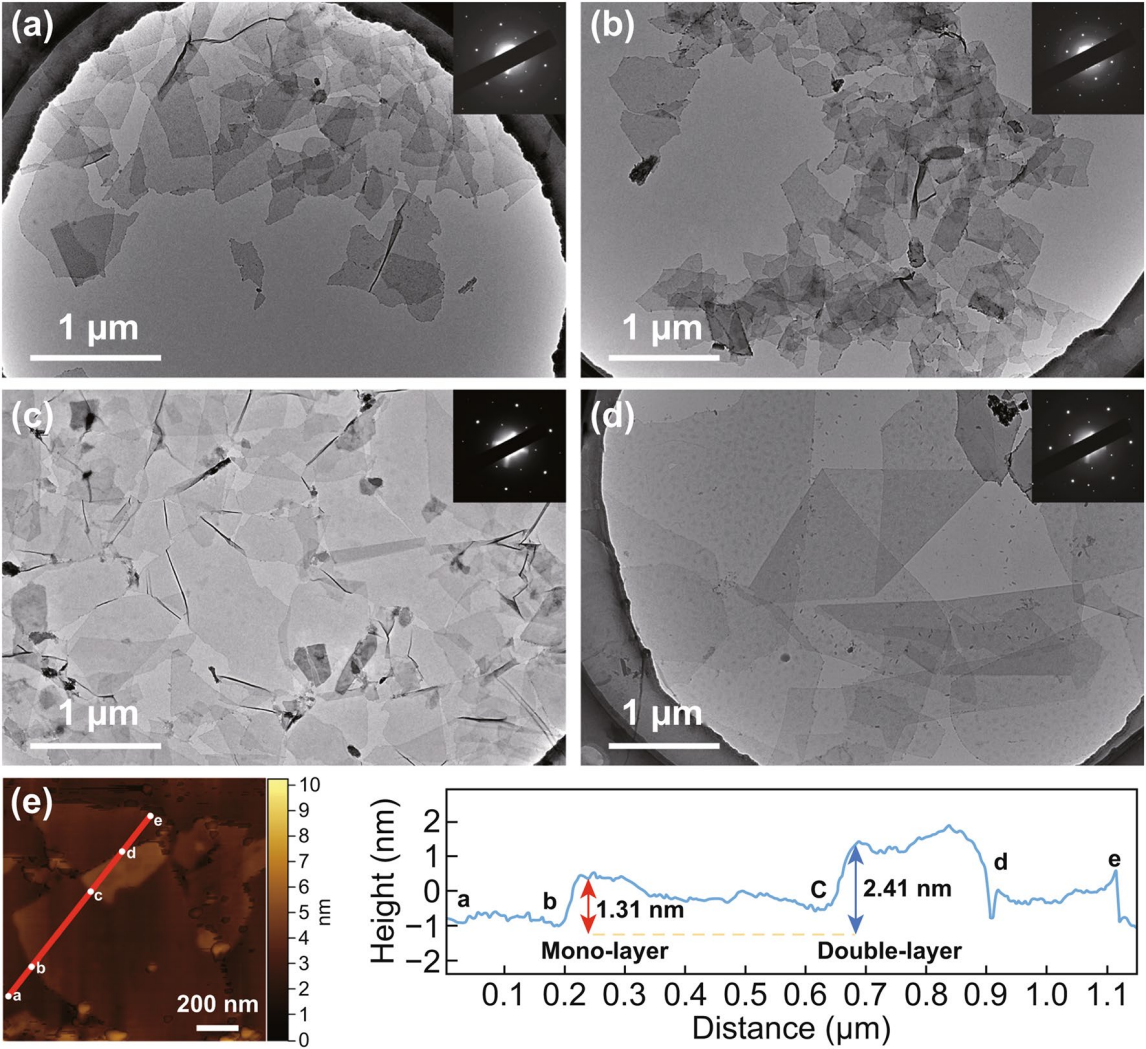
TEM images of **a** S-Ti_3_C_2_T_x_-1, **b** S-Ti_3_C_2_T_x_-6 flakes through Route II, **c** O-Ti_3_C_2_T_x_-1 and **d** O-Ti_3_C_2_T_x_-6 flakes through Route III. (Inset shows the SAED pattern). **e** AFM image of O-Ti_3_C_2_T_x_ flakes with the height profile alongside the red line

**Fig. 3:**
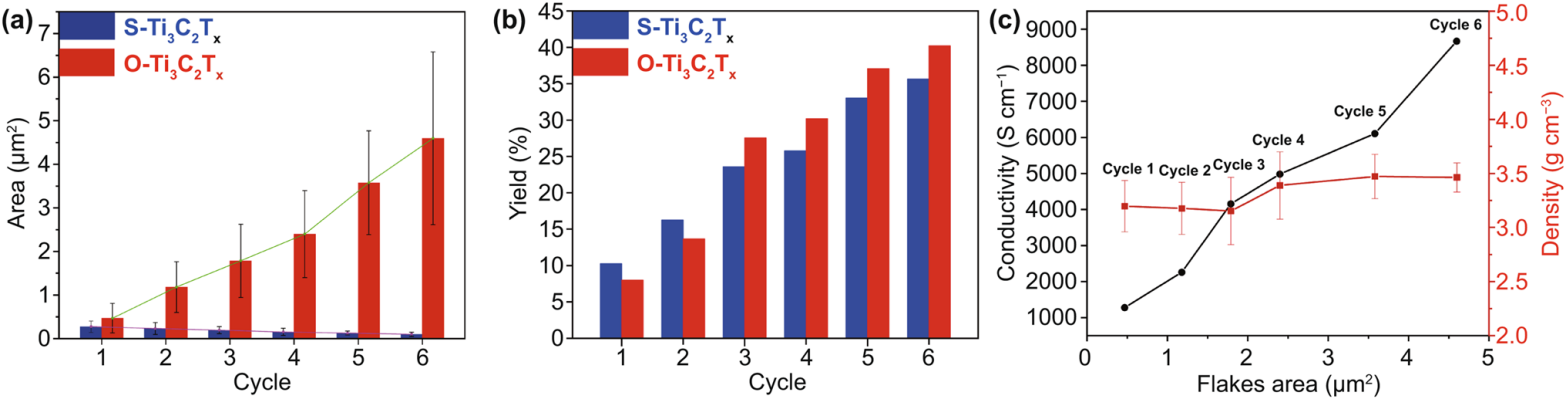
Comparison of **a** average flakes area and **b** yield at different cycles between S-Ti_3_C_2_T_x_ and O-Ti_3_C_2_T_x_. **c** Conductivity (black) and density (red) of O-Ti_3_C_2_T_x_ films versus flakes area. Cycle 1 to Cycle 6 in the figure represents the O-Ti_3_C_2_T_x_ products corresponding to different cycles

To compare the morphology of Ti_3_C_2_T_x_ flakes between Route II and Route III, Route II also carried out products harvesting in many cycles. As shown in the SEM images of the S-Ti_3_C_2_T_x_ prepared via Route II (Figs. S2a, b), S-Ti_3_C_2_T_x_ flakes are obvious 2D nanomaterials that stick together after drying. The difference in dimension of the S-Ti_3_C_2_T_x_ products obtained from the first and sixth cycle is clearly depicted in TEM images (Fig. 2a, b). It should be noted that the quality of S-Ti_3_C_2_T_x_ flakes gets worse with the increase in repeat cycles in Route II. The average area of S-Ti_3_C_2_T_x_ from different cycles gradually decreases from 0.27 to 0.10 μm^2^, as statistically analyzed in Fig. 3a. TEM images of S-Ti_3_C_2_T_x_ obtained from the other cycles are shown in Fig. S3. The SEM morphology of the O-Ti_3_C_2_T_x_-1 flakes (Fig. S2c) and O-Ti_3_C_2_T_x_-6 (Fig. S2d) prepared by the OAIC approach (Route III) is not significantly different with S-Ti_3_C_2_T_x_. However, from TEM images of O-Ti_3_C_2_T_x_-1 (Fig. 2c) and O-Ti_3_C_2_T_x_-6 (Fig. 2d), the O-Ti_3_C_2_T_x_ flakes are larger in dimension and have better quality than S-Ti_3_C_2_T_x_. The average areas of O-Ti_3_C_2_T_x_ from different cycles gradually increase from 0.47 to 4.60 μm^2^, statistically analyzed in Fig. 3a. TEM images of other cycles as shown in Fig. S4. Selected-area electron diffraction (SAED) images of O-Ti_3_C_2_T_x_ (inset in Fig. 2c, d) indicate the hexagonal crystal structure of O-Ti_3_C_2_T_x_ flakes [24], which, together with the measured *d*-spacing of 0.26 nm corresponding to the ($01\bar{1}0$) crystal plane of Ti_3_C_2_ in the high-resolution HR-TEM image (Fig. S5) indicates the successful preparation of Ti_3_C_2_T_x_ [25–27]. Atomic force microscopy (AFM) images of O-Ti_3_C_2_T_x_ flakes alongside the thickness profile are shown in Fig. 2e. From the corresponding height cutaway view, the monolayer thickness of O-Ti_3_C_2_T_x_ flake is ~ 1.31 nm, and the double-layer thickness is ~ 2.41 nm.

### Average Areas, Yield and Conductivity of Ti_3_C_2_T_x_ Products

The average flakes area (flake-area distribution diagrams in Fig. S6a) and yield of S-Ti_3_C_2_T_x_ and O-Ti_3_C_2_T_x_ products from different cycles were statistically measured and calculated. Figure 3a shows the average area of S-Ti_3_C_2_T_x_ flakes is smaller than O-Ti_3_C_2_T_x_ flakes and gradually decreases from 0.27 to 0.10 μm^2^ with different 6 cycles. The average area of O-Ti_3_C_2_T_x_ flakes is much larger than the former and gradually increases from 0.47 to 4.60 μm^2^. Therefore, as depicted in Route III in Fig. 1, to further intercalate the residues in different cycles by adding DMSO, O-Ti_3_C_2_T_x_ flakes with distinctly different dimensions could be obtained, which is the most attractive advantage of the OAIC approach. The yield of the two approaches is summarized in Fig. 3b. The total yield of O-Ti_3_C_2_T_x_ products is up to 40.2%, more than 4.6% of the S-Ti_3_C_2_T_x_ products. More importantly, the gram-level preparation of O-Ti_3_C_2_T_x_ was implemented by enlarging raw Ti_3_AlC_2_ materials and the amount of all chemical reagents. The yield of gram-level preparation of O-Ti_3_C_2_T_x_ in each cycle is listed in Table S2, and the total yield is up to 46.3%. From the TEM images for O-Ti_3_C_2_T_x_ products of gram-level preparation (Fig. S7), the dimensions of flakes also increase with different cycles and maintain good quality. Table 1 compares the lateral size, thickness and yield of O-Ti_3_C_2_T_x_ products of this work with other reports [13–17]. It is noteworthy that most reports describe the dimension of Ti_3_C_2_T_x_ flakes by lateral size, not the area. Although the area is the more accurate parameter for describing the dimension of Ti_3_C_2_T_x_ flakes, the lateral size of O-Ti_3_C_2_T_x_ flakes was also measured for comparison (Fig. S6). Therefore, the OAIC approach can prepare Ti_3_C_2_T_x_ flakes with large lateral size and simultaneously pays equal attention to the yield.

**Table 1:** Comparison of the lateral sizes, thickness, and yield of O-Ti_3_C_2_T_x_ products by OAIC approach with other reports

Method	Ti_3_AlC_2_ (mesh)	Lateral size (μm)	Thickness (nm)	Yield (%)	Refs.
FAT	200	5.52	1.6	39	[16]
HAI	200	0.3–1	1.7	74	[15]
DPS	500/400/300	0.36/0.5/0.8	/	65/57/10	[17]
BAS	/	1–4	1.2	90	[14]
MAE	/	0.8–2.2	2–4.5	< 10	[13]
OAIC	325	3.02^a^	1.31	40.2^b^	This work

^a^Average lateral size of O-Ti_3_C_2_T_x_ flakes from products of the sixth cycle

^b^Total yield of all 6 cycles

(FAT: freezing-and-thawing; HAI: hydrothermal-assisted intercalation; DPS: decreasing precursor size; BAS: binary aqueous system; MAE: microwave-assisted exfoliation; OAIC: organic solvent-assisted intercalation and collection)

The electrical conductivity is an important factor for practical applications of MXene. We prepared free-standing Ti_3_C_2_T_x_ films (Fig. S1c) through vacuum filtration for electrical conductivity measurement. The density of additive-free O-Ti_3_C_2_T_x_ films possesses densities of 3–4 g cm^−3^ [4]. Sheet resistance maps of Ti_3_C_2_T_x_ films (Fig. S6c) show small distribution of different color implies a high degree of Ti_3_C_2_T_x_ uniformity. The average sheet resistance values of O-Ti_3_C_2_T_x_-1 to O-Ti_3_C_2_T_x_-6 films listed in Fig. S6. As shown in Fig. 3c, the conductivity of O-Ti_3_C_2_T_x_ films increases with the O-Ti_3_C_2_T_x_ flake sizes, and up to 8672 S cm^−1^ of O-Ti_3_C_2_T_x_-6 with 4.06 μm^2^ flakes area. During the preparation of Ti_3_C_2_T_x_ through Route II, sonication breaks S-Ti_3_C_2_T_x_ flakes into smaller sizes and may introduce defects, which resulting in the lower electrical conductivity [19]. As shown in Figure S6, the conductivity of S-Ti_3_C_2_T_x_ film with 0.16 μm^2^ flakes area is 1062 S cm^−1^ [28], much smaller than O-Ti_3_C_2_T_x_ films. Therefore, this OAIC approach provides a means to prepare pure Ti_3_C_2_T_x_ films with good electrical conductivity by using large-size flakes.

### Chemical Composition and Structure of Ti_3_C_2_T_x_ Flakes

**Fig. 4:**
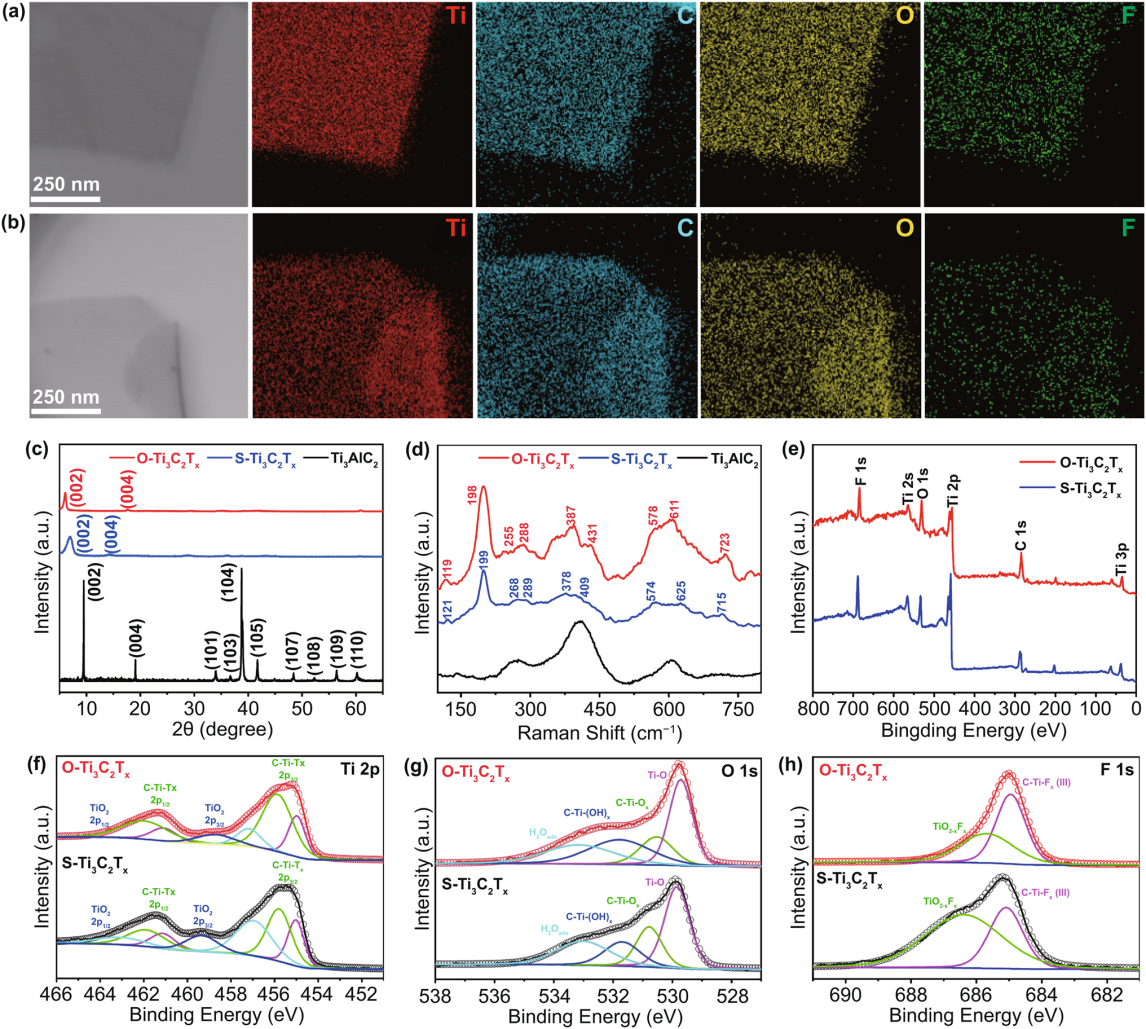
TEM images of **a** S-Ti_3_C_2_T_x_ and **b** O-Ti_3_C_2_T_x_ flakes and their corresponding elemental maps of Ti, C, O and F elements. **c** XRD patterns and **d** Raman spectra of Ti_3_AlC_2_, S-Ti_3_C_2_T_x_ and O-Ti_3_C_2_T_x_. **e** XPS survey spectra and high-resolution spectra with curve-fitting of S-Ti_3_C_2_T_x_ and O-Ti_3_C_2_T_x_ for **f** Ti 2p region, **g** O 1 s region and **h** F 1 s region

The chemical composition and structure of Ti_3_C_2_T_x_ flakes were characterized by EDS, XRD spectrum, Raman, and XPS. Figure 4a, b is the EDS element maps of S-Ti_3_C_2_T_x_ and O-Ti_3_C_2_T_x_ products, respectively, indicating that the Ti, C, O, and F elements are uniformly distributed on both S-Ti_3_C_2_T_x_ and O-Ti_3_C_2_T_x_ [24]. From the EDS spectrum of O-Ti_3_C_2_T_x_ (Fig. S8), no S element signal was detected, indicating that DMSO could be washed clean by DCM. The XRD spectra of Ti_3_AlC_2_ powders before and after LiF/HCl etching are shown in Fig. 4c. The (002) diffraction peak of Ti_3_AlC_2_ shifted to lower angles, and the intense diffraction 2*θ* peak at ≈39° of Ti_3_AlC_2_ disappeared, indicating that the Al atoms were selectively etched from the Ti_3_AlC_2_ structure [29, 30]. In addition, the (002) diffraction peak of delaminated Ti_3_C_2_T_x_ flakes shifted from 6.9° of S-Ti_3_C_2_T_x_ to 5.9° of O-Ti_3_C_2_T_x_ (Fig. S9), which corresponds to the calculated layer spacing increase from 1.28 nm of S-Ti_3_C_2_T_x_ to 1.47 nm of O-Ti_3_C_2_T_x_, demonstrating the increase in lattice space of the two products prepared by different approaches. Raman spectra (Fig. 4d) show the as-received Ti_3_AlC_2_ has similar features compared to a previous report [31], but differs from others [32, 33]. This could be due to the variation in the types of MAX products from different suppliers. Nevertheless, both A_1g_ (270 and 606 cm^−1^, referred to as the ω_4_ and ω_6_, respectively) vibrations of Ti_3_AlC_2_ shift as a result of etching and Ti_3_C_2_T_x_ formation. In the Raman spectra of Ti_3_C_2_T_x_ products, the prominent peak ~ 200 cm^−1^ is assigned to the out-of-plane (A_1g_) vibration of Ti, O, and C atoms, and ~ 720 cm^−1^ to another A_1g_ vibration of C atoms. The region around 230–470 and 500–650 cm^−1^ can be assigned to in-plane (E_g_) vibrations of surface groups [34]. As in a previous study, the higher intensity of peaks at 200, 387, 611, and 723 cm^−1^ for O-Ti_3_C_2_T_x_ flakes compared with S-Ti_3_C_2_T_x_ flakes might be attributed to the differences in flake sizes and surface functional groups between the two approaches [19]. The surficial structural evidence of Ti_3_C_2_T_x_ flakes was investigated by XPS. The survey spectrum (Fig. 4e) shows that no S 2p peak (165.5 eV) was detected in O-Ti_3_C_2_T_x_ products, which proves that no DMSO residues were left after washing with DCM by the OAIC approach. The atomic ratios of each element from the XPS data of S-Ti_3_C_2_T_x_ and O-Ti_3_C_2_T_x_ are shown in Table S1, which is consistent with the literature reports [35]. High-resolution XPS spectra provide different surficial structural evidences between S-Ti_3_C_2_T_x_ and O-Ti_3_C_2_T_x_. The Ti 2p high-resolution XPS spectrum (Fig. 4f) shows that the signals at 455.8 and 462.1 eV (marked as C-Ti-T_x_ 2p_3/2_ and C-Ti-T_x_ 2p_1/2_, respectively) correspond to Ti-C bonds, and the signals at 459.4 and 465.7 eV (marked as TiO_2_ 2p_3/2_ and TiO_2_ 2p_1/2_, respectively) correspond to Ti–O bonds [36]. It is noteworthy that from S-Ti_3_C_2_T_x_ to O-Ti_3_C_2_T_x_ (as below), there is a distinct increase and decrease in the signal intensity at C-Ti-T_x_ 2p_3/2_ and TiO_2_ 2p_3/2_, respectively, indicating that there is less oxidation of O-Ti_3_C_2_T_x_ than S-Ti_3_C_2_T_x_ [20]. In the O 1 s high-resolution spectrum (Fig. 4g), the intensity of C-Ti-O_x_ signal (~ 530.8 eV) decreases, indicating greater amounts of -OH groups on O-Ti_3_C_2_T_x_ than on -O groups. In addition, a significantly decrease in the intensity of TiO_2-x_F_x_ signal (≈685.7 eV, Fig. 4h), indicating less -F groups on the surface of O-Ti_3_C_2_T_x_ [35]. The absorption spectra between 300 and 1100 nm (UV–Vis) are shown in Figure S10. Both S-Ti_3_C_2_T_x_ and O-Ti_3_C_2_T_x_ spectra show strong characteristic Ti_3_C_2_T_x_ absorbance peaks at ~ 320 and ~ 770 nm, respectively [28, 37–39]. After six-time cycles, there was no significant change at the intensity peak of O-Ti_3_C_2_T_x_, indicating no obvious chemical oxidation during the OAIC approach.

### Electrochemical Performance of Ti_3_C_2_T_x_ Products

**Fig. 5:**
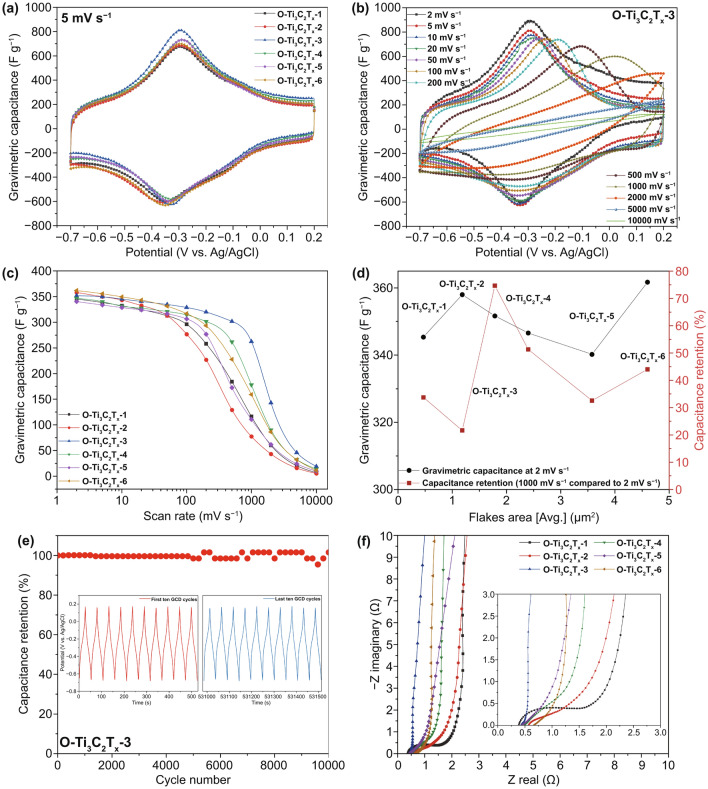
Electrochemical performance of O-Ti_3_C_2_T_x_ film electrodes. **a** Cyclic voltammogram profiles collected in 3 M H_2_SO_4_ at 5 mV/s scan rate of O-Ti_3_C_2_T_x_ film electrodes from products of different cycles (denote as O-Ti_3_C_2_T_x_-1 to O-Ti_3_C_2_T_x_-6). **b** CV profiles of O-Ti_3_C_2_T_x_-3 at scan rates from 2 mV s^−1^ to 10 V s^−1^. **c** Gravimetric capacitance of O-Ti_3_C_2_T_x_ film electrodes at different scan rates **d** Gravimetric capacitance at scan rate of 2 mV s^−1^ and rate performance (scan rate at 1000 mV s^−1^ compared to at 2 mV s^−1^) of O-Ti_3_C_2_T_x_ film electrodes versus different flakes areas. **e** Capacitance stability of O-Ti_3_C_2_T_x_-3 performed by GCD cycling at 10 A g^−1^. The insets depict GCD cycling profiles collected at first ten cycles (red) and last ten cycles (blue), respectively. **f** Electrochemical impedance spectroscopy (EIS) for O-Ti_3_C_2_T_x_ film electrodes. The inset shows a magnification of the high-frequency region

To study the electrochemical performance of the O-Ti_3_C_2_T_x_ products by OAIC approach, we vacuum-filtered the solutions of O-Ti_3_C_2_T_x_ products of different cycles to form free-standing Ti_3_C_2_T_x_ films (Fig. S1c). The electrochemical measurements were conducted by a three-electrode system in a Swagelok cell (Fig. S1d, e) with O-Ti_3_C_2_T_x_ films as working electrodes in an electrochemical capacitor. Cyclic voltammetry (CV) plots of the O-Ti_3_C_2_T_x_ film electrodes at 5 mV s^−1^ (Fig. 5a) show appearance of redox peaks associating with the small cathodic and anodic peak potential separation (less than 50 mV), indicating that O-Ti_3_C_2_T_x_ conforms with the pseudocapacitive charge storage behavior [28, 40, 41]. The galvanostatic charge–discharge (GCD) profiles show symmetric triangular curves at varied current densities, indicating fast and reversible electrochemical reactions (Fig. S11). Specific capacitances were calculated by integration of the discharge curves in the CV plots (Figs. 5b and S12) of different O-Ti_3_C_2_T_x_ film electrodes. At a scan rate of 2 mV s^−1^, gravimetric capacitance values of all O-Ti_3_C_2_T_x_ products from differernt cycles are more than 340 F g^−1^ (Fig. 5c), and volumetric capacitance values are up to 1100 F cm^−3^ (Fig. S13). A good rate performance was also observed, specifically, the O-Ti_3_C_2_T_x_-3 outperformed others with a capacitance retention of 75% (at scan rate of 1000 mV s^−1^ compare to 2 mV s^−1^, Fig. 5d). We also investigated the capacitive performance of S-Ti_3_C_2_T_x_. As shown in Fig. S14, the gravimetric capacitance of S-Ti_3_C_2_T_x_ is ~ 300 F g^−1^, which similarly with prsitine Ti_3_C_2_T_x_ of previous reports [12, 18, 28, 42–45]. The gravimetric capacitances and rate performance of O-Ti_3_C_2_T_x_ film electrodes were compared with pristine Ti_3_C_2_T_x_ of previous reports and summarized in Table S3, which clearly demonstrate that O-Ti_3_C_2_T_x_ products show outstanding capacitive performance. Using the best rate performing products of O-Ti_3_C_2_T_x_-3, the cyclic performance was also investigated. As shown in Fig. 5e, the O-Ti_3_C_2_T_x_-3 film electrode shows almost a 100% capacitance retention after 10,000 cycles at a GCD rate of 10 A g^−1^.

In a previous work, Gogotsi et al. studied the size-dependent electrochemical properties of Ti_3_C_2_T_x_ flakes [28]. For obtaining Ti_3_C_2_T_x_ flakes with different sizes, they used solution-processable techniques to control and sort Ti_3_C_2_T_x_ flakes after synthesis based on sonication and sucrose density gradient centrifugation, respectively. As one of the attractive features of this OAIC approach, the Ti_3_C_2_T_x_ flakes with different sizes can be obtained in different production cycles instead of sonication and density gradient centrifugation, in order that provide an alternative approach to investigate the size-dependent electrochemical properties of Ti_3_C_2_T_x_ flakes. We summarized the gravimetric capacitance (scan rate at 2 mV s^−1^) and capacitance retention (scan rate at 1000 mV s^−1^ compared to at 2 mV s^−1^) of O-Ti_3_C_2_T_x_ film electrodes with different flakes areas (corresponding flakes lateral sizes are shown in Fig. S6) in Fig. 5d. The O-Ti_3_C_2_T_x_-6 with largest flakes area shows the best specific capacitance of 362 F g^−1^ (also for volumetric capacitance which is 1253 F cm^−3^) at the scan rate of 2 mV s^−1^. However, from the perspective of the rate performance, the O-Ti_3_C_2_T_x_-3 with median flakes area outperforms others. The size-dependent electrochemical properties of Ti_3_C_2_T_x_ have a compromise between the electrical conductivity and ion pathways of electrolyte. Specifically, although the larger Ti_3_C_2_T_x_ flakes have higher electrical conductivity which heighten the charge transfer thereby boosting the capacitive performance [28], the film electrodes prepared by larger Ti_3_C_2_T_x_ flakes suffer from long ion transport pathways resulting the poor rate performance [42]. Therefore, the O-Ti_3_C_2_T_x_-3 with median flakes area finds a balance between the good electrical conductivity and the less hinder of ion transport of electrolyte. The electrochemical impedance spectra (EIS) of O-Ti_3_C_2_T_x_-1 to O-Ti_3_C_2_T_x_-6 film electrodes are shown in Fig. 5f, which is highly consistent with the electrical conductivity and capacitance performance. From the high-frequency region in Fig. 5f, the O-Ti_3_C_2_T_x_-1 and O-Ti_3_C_2_T_x_-2 possess a comparatively larger diameter of semicircle arc which related to charge transfer resistance (R_ct_) due to the lower electrical conductivity. From O-Ti_3_C_2_T_x_-3 to O-Ti_3_C_2_T_x_-6, the Nyquist plots have relatively lower R_ct_ corresponding to the good electrical conductivity [28, 46]. The 45° slopes of the O-Ti_3_C_2_T_x_-3 are shorter than others, indicating the shorter ion transport pathways [28]. The straight line of O-Ti_3_C_2_T_x_-3 is almost parallel to the imaginary axis, indicating the rapid ion diffusion corresponding to the good rate performance [42, 45].

## Conclusions

In summary, a new approach (OAIC) developed to prepare Ti_3_C_2_T_x_ MXene has several remarkable features, including gram-level preparation with high yield (~ 46.3%), controllable flakes size (0.47–4.60 μm^2^), higher safety (toxic HF free), a good electrical conductivity (8,672 S cm^−1^), an outstanding capacitive performance (352 F g^−1^), and less requirement for facilities (high-speed centrifugation-free). The morphology, crystal phase, chemical bonds and elemental information of the products have been thoroughly studied by SEM/TEM, XRD/SAED, Raman spectrum/XPS, and EDS, respectively. To our knowledge, this novel OAIC approach makes a good trade-off between laboratory operation, requirement for facility, and product quality, which not only gives an excellent example regarding the synthesis of other MXene materials, but also sheds new light on the mass production of Ti_3_C_2_T_x_ MXene in the future.

## Supplementary Information

Below is the link to the electronic supplementary material.

Supplementary file1 (PDF 1543 kb)

Supplementary file2 (MP4 5551 kb)
