# Supplementary material for: An Organic Solvent-Assisted Intercalation and Collection (OAIC) for Ti3C2Tx MXene with Controllable Sizes and Improved Yield
Source: Nanomicro Lett. 2021 Sep 5;13:188. doi: 10.1007/s40820-021-00705-4 (PMC8418585; doi:10.1007/s40820-021-00705-4)
Supplement: Supplementary file 1 — Supplementary file1 (PDF 1543 kb) [file 40820_2021_705_MOESM1_ESM.pdf]

Supporting Information for

Organic Solvent Assisted Intercalation and Collection for $Ti_3C_2T_x$ MXene with Controllable Sizes and Improved Yield

Danyao Qu[1], Yingying Jian[1], Lihao Guo[1], Chen Su[1], Ning Tang[2], Xingmao Zhang[1], Wenwen Hu[2], Zheng Wang[1], Zhenhuan Zhao[1], Peng Zhong[1], Peipei Li[1], Tao Du[1, *], Hossam Haick[3, *], and Weiwei Wu[1, *]

[1]School of Advanced Materials and Nanotechnology, Xidian University, Xi'an, Shaanxi 710126, P. R. China

[2]School of Aerospace Science and Technology, Xidian University, Xi'an, Shaanxi 710126, P. R. China

[3]Department of Chemical Engineering and Russell Berrie Nanotechnology Institute, Technion-Israel Institute of Technology, Haifa 3200003, Israel

*Corresponding authors. E-mail: taodu@xidian.edu.cn (Tao Du); hhossam@technion.ac.il (Hossam Haick); wwwu@xidian.edu.cn (Weiwei Wu)

Supplementary Tables and Figures

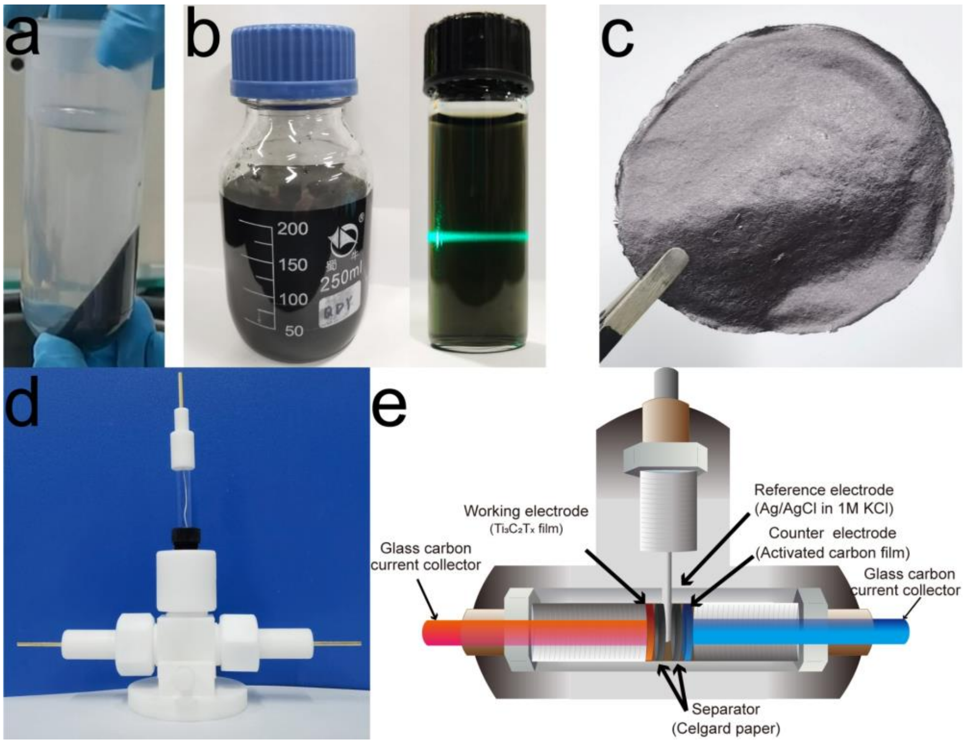

Fig. S1 Digital photographs of **(a)** supernatant and sediment after adding DCM and centrifugation at speed of 2,000 rpm (Step 6). **(b)** the synthesized O-$Ti_3C_2T_x$ solution placed for one week at room temperature (left) and the diluted solution showing Tyndall

scattering effect (right). **(c)** the O-$Ti_3C_2T_x$ film with diameter 40 mm made by vacuum-assisted filtration, indicating the good film-forming ability. **(d)** the Swagelok three-electrode setup used in electrochemical measurements. **(e)** Schematic representation of a Swagelok cell. The glassy carbon electrodes were used as current collectors for both the working and the counter electrodes. The prepared $Ti_3C_2T_x$ films were punched to the desired size and were directly used as the working electrode without addition of any binder. The freestanding overcapactive activated carbon electrode was used as the counter electrode. The Ag/AgCl electrode in 1 M KCl was used as the reference electrode. Two pieces of Celgard paper (3501) were used as the separator between the working electrode and the counter electrode. The deaerated 3 M H_2SO_4 was used as the electrolyte.

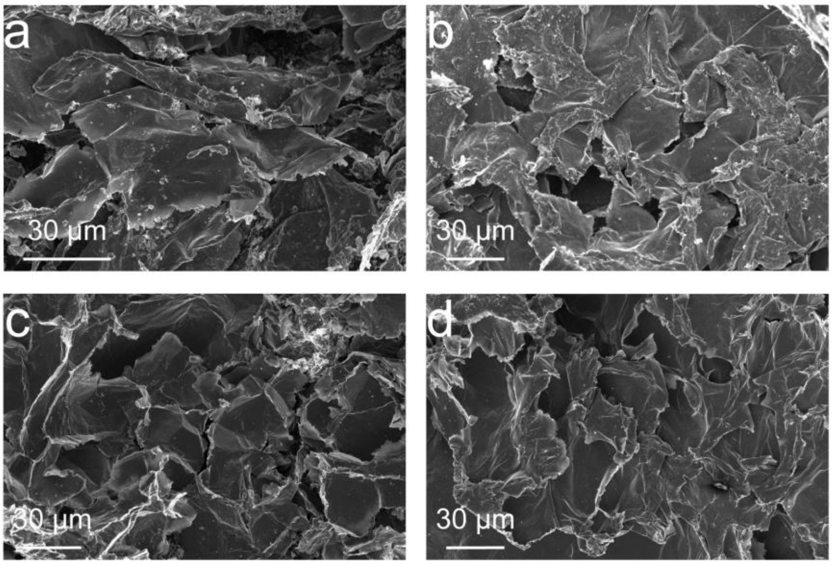

Fig. S2 SEM images of **(a)** S-$Ti_3C_2T_x$-1, **(b)** S-$Ti_3C_2T_x$-6 flakes through Route II, and **(c)** O-$Ti_3C_2T_x$-1, **(d)** O-$Ti_3C_2T_x$-6 flakes through Route III

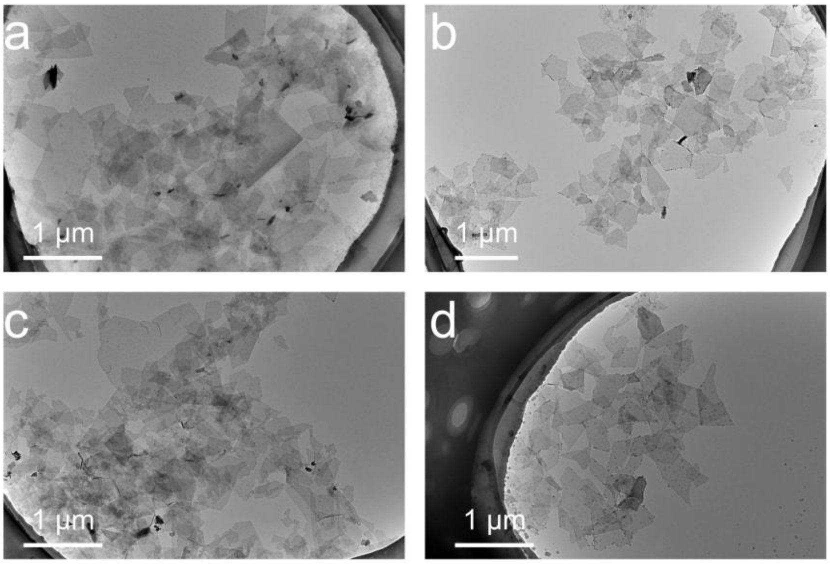

Fig. S3 TEM images of **(a)** S-$Ti_3C_2T_x$-2, **(b)** S-$Ti_3C_2T_x$-3, **(c)** S-$Ti_3C_2T_x$-4, **(d)** S-$Ti_3C_2T_x$-5 flakes

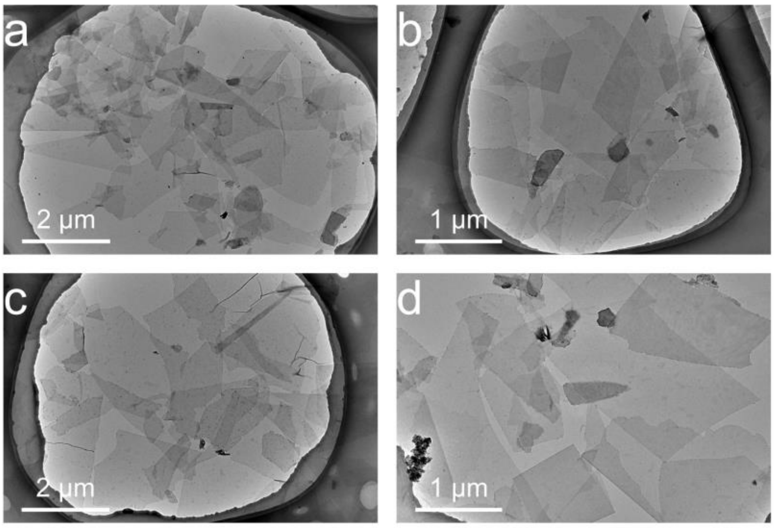

Fig. S4 TEM images of **(a)** O-$Ti_3C_2T_x$-2, **(b)** O-$Ti_3C_2T_x$-3, **(c)** O-$Ti_3C_2T_x$-4, (**d**) O-$Ti_3C_2T_x$-5 flakes

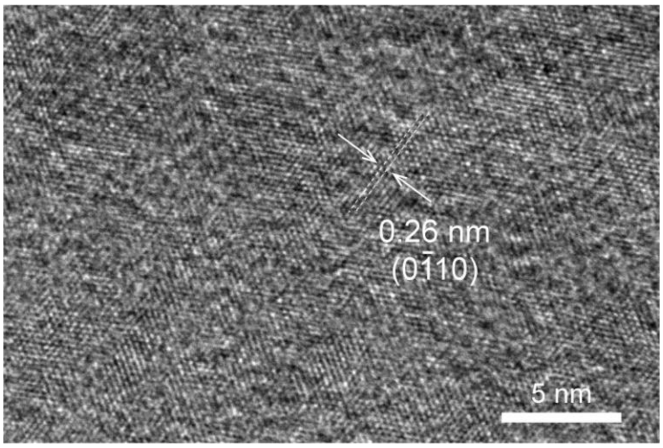

Fig. S5 High-resolution TEM image of O-$Ti_3C_2T_x$

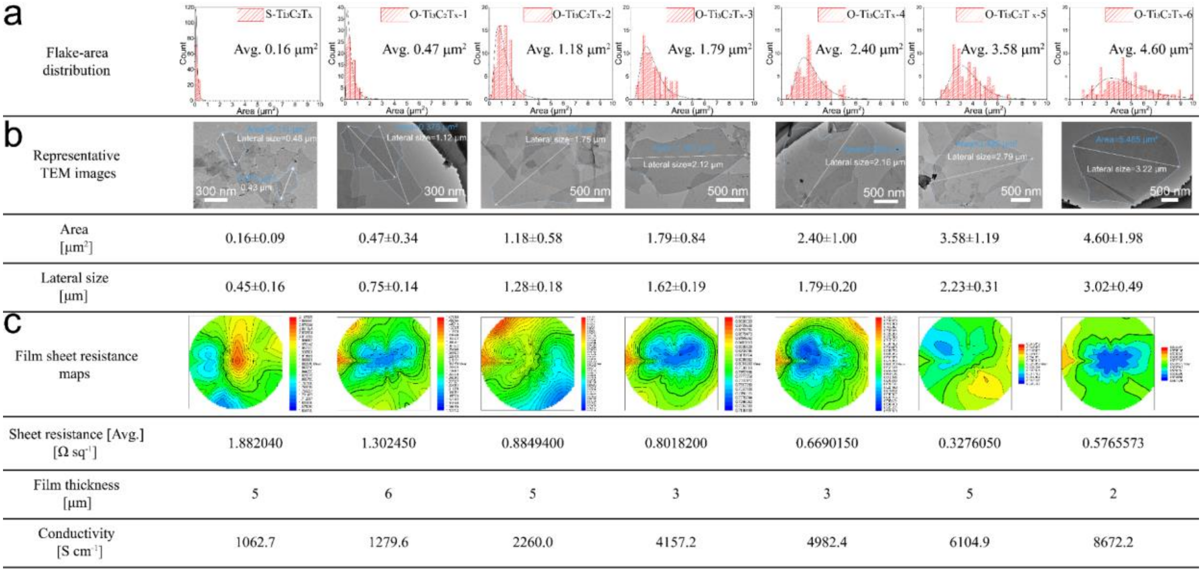

Fig. S6 S-$Ti_3C_2T_x$ and O-$Ti_3C_2T_x$ flakes size, structure, and conductivity. **(a)** Flake-area distribution diagrams, **(b)** Representative TEM images of $Ti_3C_2T_x$ flakes. Blue lines

outline the area of $Ti_3C_2T_x$ flakes, and white lines outline the lateral size of $Ti_3C_2T_x$ flakes. TEM-derived area and lateral size distributions were determined by measuring the area and lateral size of 100 flakes. The average values of flakes area and lateral size list below. (**c**) Sheet resistance (Rs) maps of $Ti_3C_2T_x$ films fabricated by vacuum filtration. The average values of Rs and corresponding film thickness (**d**) list below. The conductivity, σ (S cm^{-1}), was calculated by σ=1/(Rs×d)

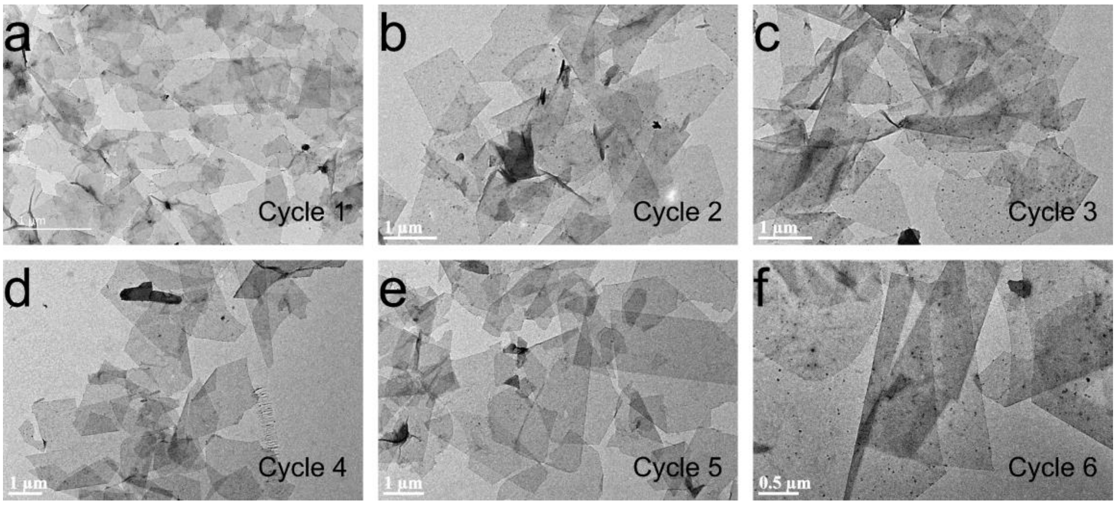

Fig. S7 TEM images for O-$Ti_3C_2T_x$-1 to O-$Ti_3C_2T_x$-6 flakes of gram-level preparation

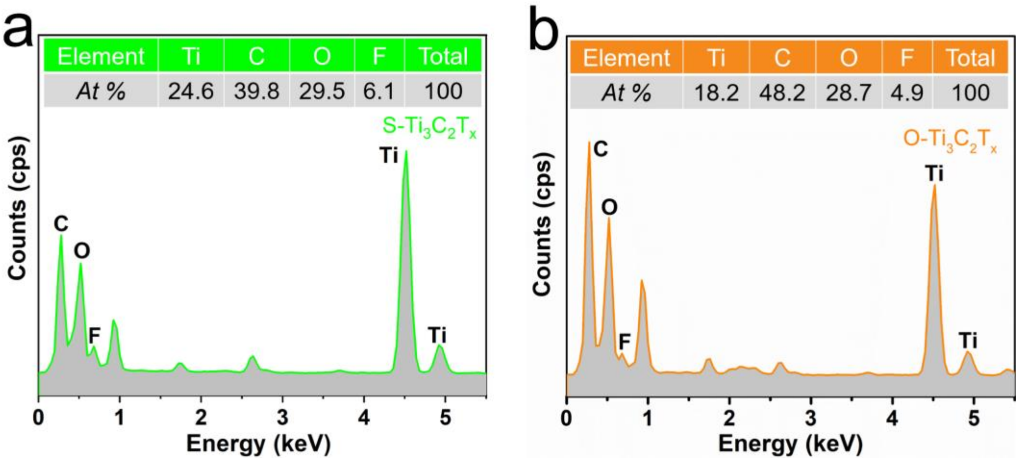

Fig. S8 Energy-dispersive X-ray spectroscopy (EDS) spectra of (**a**) S-$Ti_3C_2T_x$ and (**b**) O-$Ti_3C_2T_x$

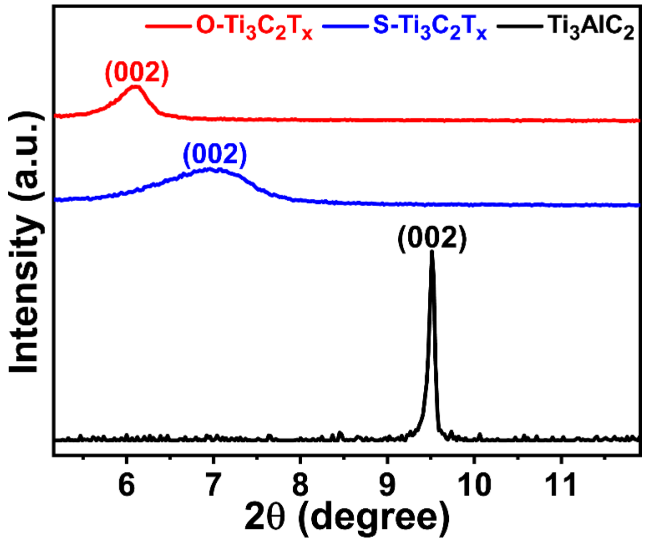

Fig. S9 Magnifying XRD patterns in Fig. 3c

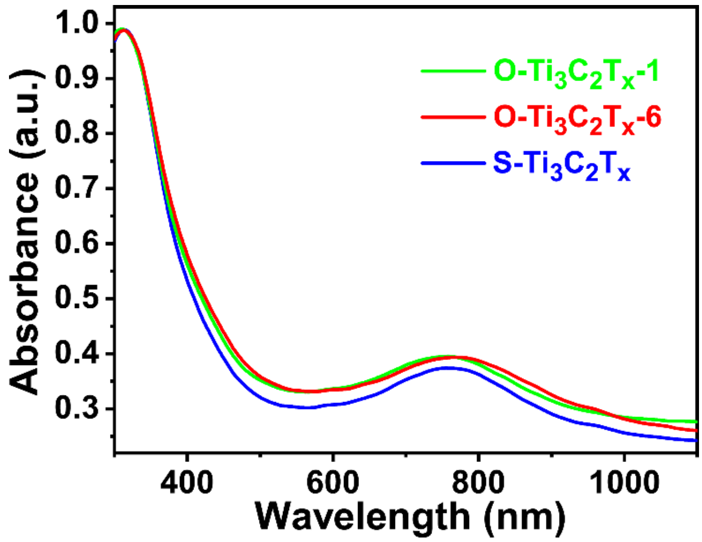

Fig. S10 UV-vis spectra of S-$Ti_3C_2T_x$ and O-$Ti_3C_2T_x$

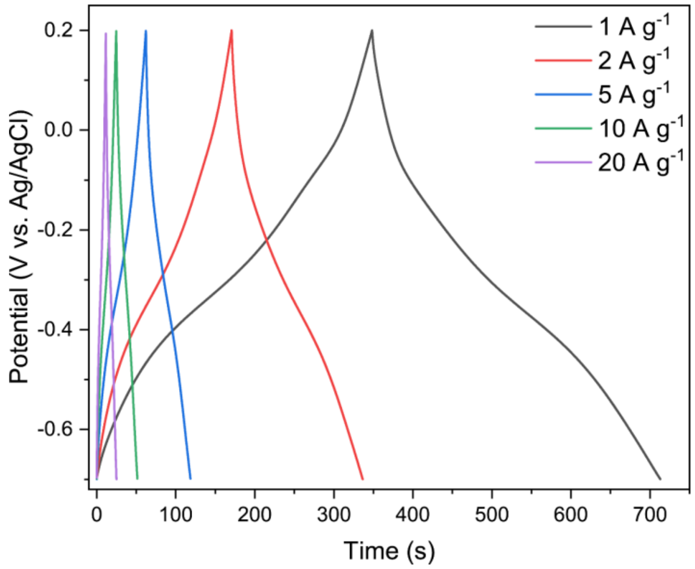

Fig. S11 Galvanostatic charge-discharge (GCD) profiles of O-$Ti_3C_2T_x$-3

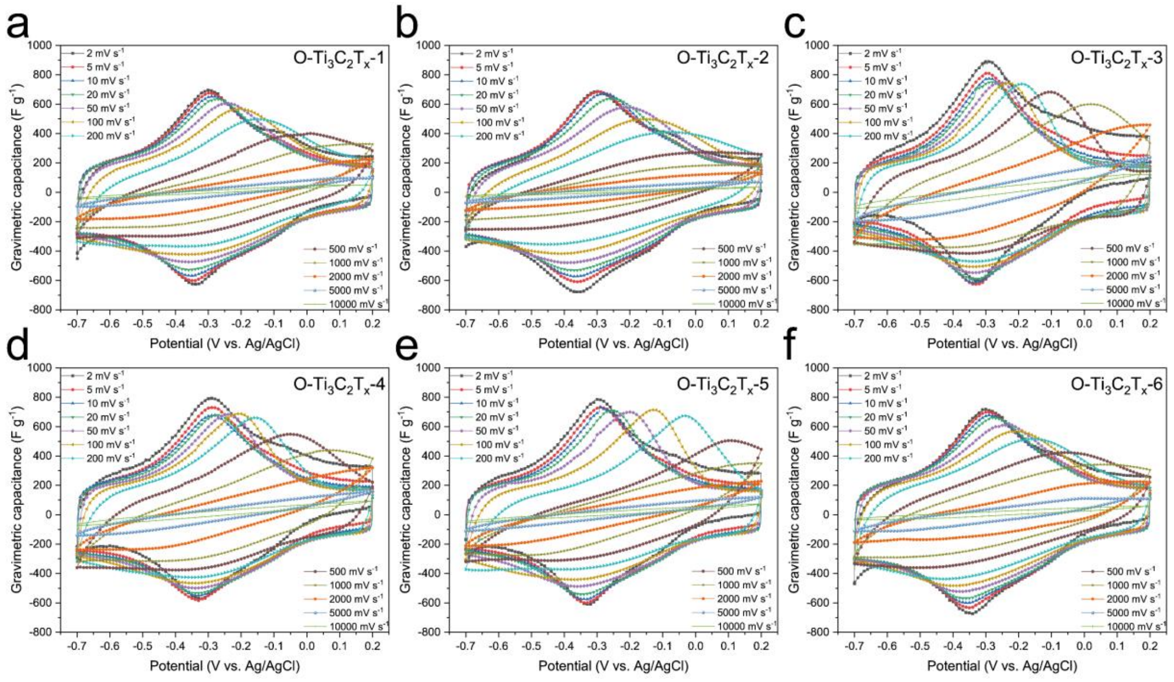

Fig. S12 CV profiles of O-$Ti_3C_2T_x$-1 to O-$Ti_3C_2T_x$-6 film electrodes at scan rates from 2 mV s^{-1} to 10 V s^{-1}

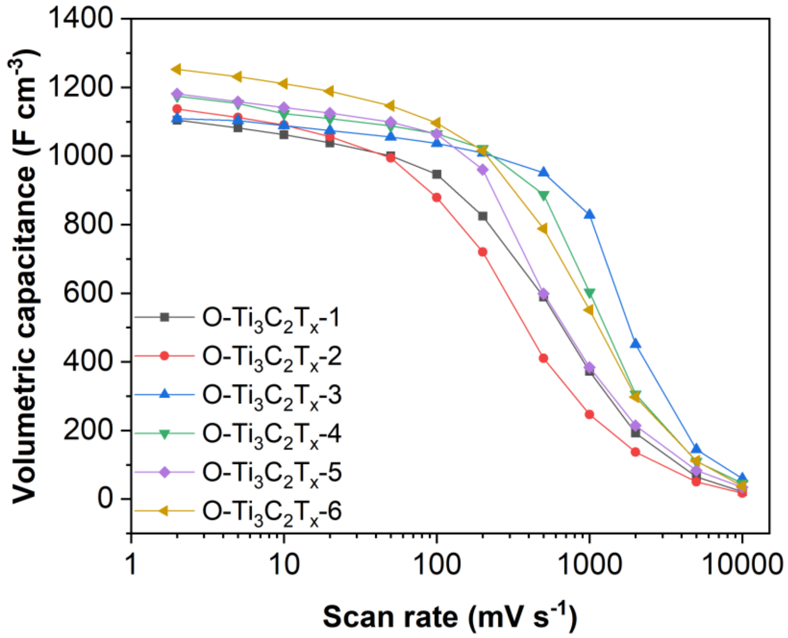

Fig. S13 Volumetric capacitance of O-$Ti_3C_2T_x$-1 to O-$Ti_3C_2T_x$-6 film electrodes at different scan rates

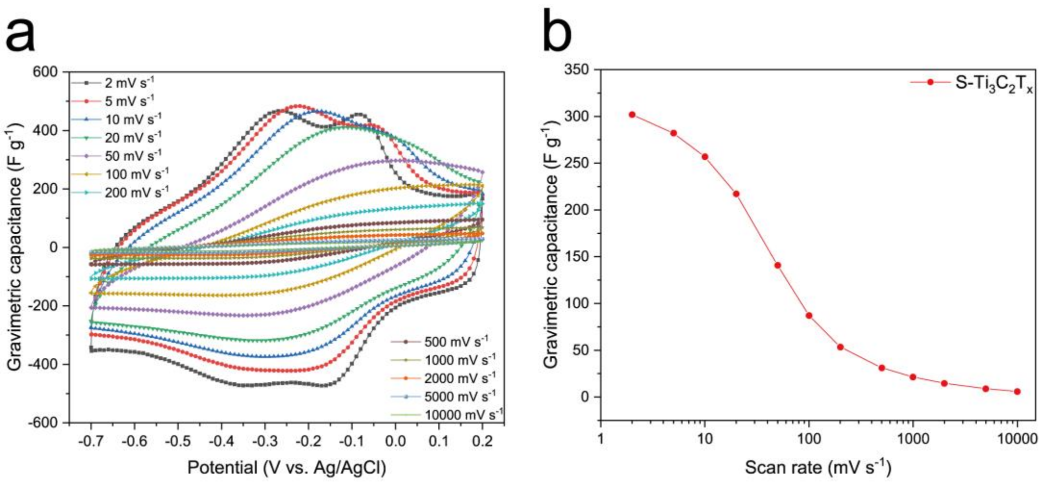

Fig. S14 (**a**) CV profiles of S-$Ti_3C_2T_x$, (**b**) Gravimetric capacitance of S-$Ti_3C_2T_x$ film electrode at different scan rates

Table S1 Summary of atomic ratios of S-$Ti_3C_2T_x$ and O-$Ti_3C_2T_x$

	Ti (at%)	C (at%)	O (at%)	F (at%)
S-$Ti_3C_2T_x$	29.22	40.69	17.00	13.09
O-$Ti_3C_2T_x$	30.12	45.89	15.19	8.80

Table S2 Yield calculation of gram-level preparation for O-$Ti_3C_2T_x$

Cycle	m (g)	$\bar{m}$ (g)	c (g mL^{-1})	m_{dis} (g)	Total production (g)
1	m_1=0.0547<br>m_2=0.0533<br>m_3=0.0534<br>m_4=0.0533<br>m_5=0.0533	0.0536	0.01072	1.2864	4.62928<br>Yield = 46.29 %
2	m_1=0.0480<br>m_2=0.0479<br>m_3=0.0477<br>m_4=0.0480<br>m_5=0.0478	0.04788	0.009576	0.76608	
3	m_1=0.0276<br>m_2=0.0268<br>m_3=0.0281<br>m_4=0.0276<br>m_5=0.0278	0.02758	0.005516	1.54448	
4	m_1=0.0230<br>m_2=0.0230<br>m_3=0.0230<br>m_4=0.0231<br>m_5=0.0230	0.02302	0.004604	0.82872	
5	m_1=0.0056	0.00562	0.001124	0.1124	

	m_2=0.0055<br>m_3=0.0056<br>m_4=0.0057<br>m_5=0.0057				
6	m_1=0.0046<br>m_2=0.0044<br>m_3=0.0044<br>m_4=0.0046<br>m_5=0.0048	0.00456	0.000912	0.0912	

The volumes of dispersion in step 8 in each cycle were accurately measured which denoted as V_{dis}. Then, the dispersion of each cycle was extracted for five parts with 5 ml of each part. After freeze-drying, the O-$Ti_3C_2T_x$ products of each part were weighted and denoted as m_1 to m_5. The mass concentration of the dispersion can be calculated by the following equation: $c = \frac{\bar{m}}{V}$, where $\bar{m}$ is the average mass of five parts, V is 5 ml. So far, the mass of O-$Ti_3C_2T_x$ products in dispersion of each cycle (denote as m_{dis}) can be calculated by the following equation: $m_{dis} = cV_{dis}$. The yield of gram-level preparation was calculated by total mass of O-$Ti_3C_2T_x$ products in the dispersion dividing by the mass of Ti_3AlC_2 powders (10 g).

Table S3 A brief summary of capacitive performance of pristine $Ti_3C_2T_x$

Materials	Synthesis methods	Lateral Size	Electrolyte	Gravimetric capacitance	Rate performance [a]	Refs.
S-pristine $Ti_3C_2T_x$	HCl/HF	150 nm	3M H_2SO_4	~300 F g^{-1}@ 5 mV s^{-1}	~70% at 1000mV s^{-1}	[S1]
L-pristine $Ti_3C_2T_x$	HCl/HF	1.28 μm	3M H_2SO_4	~290 F g^{-1}@ 5 mV s^{-1}	~20.7% at 1000 mV s^{-1}	[S1]
Pristine $Ti_3C_2T_x$	HCl/LiF	< 1 μm	3M H_2SO_4	348 F g^{-1}@ 5 mV s^{-1}	~30.2% at 1000 mV s^{-1}	[S2]
Pure $Ti_3C_2T_x$	HCl/LiF	/	3M H_2SO_4	303 F g^{-1}@ 2 mV s^{-1}	34% at 500 mV s^{-1}	[S3]
Pure $Ti_3C_2T_x$	HCl/LiF	0.5 - 1.5 μm	1M H_2SO_4	245 F g^{-1}@ 2 mV s^{-1}	~83% at 100 mV s^{-1}	[S4]
Pure $Ti_3C_2T_x$	HCl/LiF	~1 μm	3M H_2SO_4	290 F g^{-1}@ 2 mV s^{-1}	69% at 1000 mV s^{-1}	[S5]
Pure $Ti_3C_2T_x$	HCl/LiF	~200-300 nm	3M H_2SO_4	~260 F g^{-1}@ 10 mV s^{-1}	~60% at 1000 mV s^{-1}	[S6]
Pristine $Ti_3C_2T_x$	HCl/HF	/	1M H_2SO_4	~300 F g^{-1}@ 5 mV s^{-1}	~43.3% at 1000 mV s^{-1}	[S7]
O-$Ti_3C_2T_x$-3	OAIC	1.62 μm	3M H_2SO_4	352 F g^{-1}@ 2 mV s^{-1}	74.7% at 1000 mV s^{-1}	This work

[a]. Rate performance was collectted by reported data or calcultated according to gravimetric capacitance at the indicated scan rate compared to the values in column 5.

Supplementary References

[S1] J. Tang, T. Mathis, X. Zhong, X. Xiao, H. Wang et al., Optimizing ion pathway in titanium carbide MXene for practical high-rate supercapacitor. Adv. Energy Mater. **11**, 2003025 (2021). https://doi.org/10.1002/aenm.202003025

[S2] W. Tian, A. VahidMohammadi, M.S. Reid, Z. Wang, L. Ouyang et al., Multifunctional nanocomposites with high strength and capacitance using 2D MXene and 1D nanocellulose. Adv. Mater. **0**, 1902977 (2019). https://doi.org/10.1002/adma.201902977

[S3] Z. Fan, Y. Wang, Z. Xie, D. Wang, Y. Yuan et al., Modified MXene/holey graphene films for advanced supercapacitor electrodes with superior energy storage. Adv. Sci. **5**, 1800750 (2018). https://doi.org/10.1002/advs.201800750

[S4] M. Ghidiu, M.R. Lukatskaya, M.-Q. Zhao, Y. Gogotsi, M.W. Barsoum, Conductive two-dimensional titanium carbide ‘clay’ with high volumetric capacitance. Nature **516**, 78-81 (2014). https://doi.org/10.1038/nature13970

[S5] K. Maleski, C.E. Ren, M.-Q. Zhao, B. Anasori, Y. Gogotsi, Size-dependent physical and electrochemical properties of two-dimensional MXene flakes. ACS Appl. Mater. Interfaces **10**, 24491-24498 (2018). https://doi.org/10.1021/acsami.8b04662

[S6] Y. Xia, T.S. Mathis, M.-Q. Zhao, B. Anasori, A. Dang et al., Thickness-independent capacitance of vertically aligned liquid-crystalline MXenes. Nature **557**, 409-412 (2018). https://doi.org/10.1038/s41586-018-0109-z

[S7] J. Tang, T.S. Mathis, N. Kurra, A. Sarycheva, X. Xiao et al., Tuning the electrochemical performance of titanium carbide MXene by controllable in situ anodic oxidation. Angew. Chem. Int. Ed. **58**, 17849-17855 (2019). https://doi.org/10.1002/anie.201911604
